# Personalized Dentistry: Approaching a New Way for Diagnosis and Treatment of Oral Diseases

**DOI:** 10.3390/jpm10020035

**Published:** 2020-05-01

**Authors:** Romeo Patini

**Affiliations:** Department of Head, Neck and Sense Organs, School of Dentistry, Fondazione Policlinico Universitario A. Gemelli IRCCS, Università Cattolica del Sacro Cuore, 00168 Rome, Italy; romeo.patini@unicatt.it

**Keywords:** oral microbiota, personalized dentistry, systemic diseases, clinical trial, systematic review

## Abstract

For years, it has been thought that the field of dentistry was referring exclusively to some diseases that strictly affect the oral cavity. Dental caries, periodontal disease, and pathologies associated with their worsening were considered almost the only interest in scientific research in dentistry. Recent studies have begun to shed light on the effect of the oral microbiota on general health and on the crucial role of dentistry in its maintenance. In this way, we came to understand that the bacterial populations that make up the oral microbiota can vary profoundly between individuals and that contribute in a fundamental way to outlining the so-called “oral signature”. This characteristic is called into question to evaluate the susceptibility, or lack thereof, of the subject to the contraction of a wide range of pathologies, apparently not connected with oral health. From this evidence, it will also be possible to study therapeutic approaches aimed at the eradication of species considered at risk or colonization with species considered protective; thus, giving life to so-called “personalized dentistry”. Therefore, this *Special Issue* is aimed at spreading the scientific knowledge over the current limits in terms of new molecular and culturomic approaches towards the diagnosis of oral microbiota and the treatment techniques of eventually associated systemic diseases. In vivo studies and systematic literature reviews with quantitative analysis of results, when possible, will be given a high priority.

The majority of the most common oral diseases are chronic infectious-inflammatory diseases affecting the dental elements: supporting tissues or lining ones. In fact, caries and periodontal disease alone represent over 90% of all diseases of the oral cavity [1]. The remaining part is represented by lesions often connected to aggravations of the aforementioned pathologies, such as pulpits, abscesses, empyema, or phlegmons. Nonetheless, we are also witnessing a worrying growth in cases of oncological pathologies of the oral cavity, inevitably linked to the economic crisis, which has significantly reduced access to care for less well-off patients. The reduction of preventive and control dental visits has, therefore, strongly reduced the early diagnosis of both infectious-inflammatory and oncological lesions.

Recent evidence has linked the onset of some types of oral cancer to the increase in the prevalence of some microbial populations [2,3,4], whereas such evidence has been known for years in regards to caries, periodontitis, and other diseases related to their aggravation [5,6,7,8,9,10,11,12,13,14,15,16,17].

Until the early 2000s, it was believed that few bacteria could originate pathological processes affecting periodontium [18]. Although it was believed that these species were few; however, their analysis was not always as simple as the collection of plaque samples, as it was a difficult procedure to perform while avoiding contamination. Additionally, the analysis methods did not allow adequate recognition of all species, and the data analysis software could not make associations with overly large data sets [19].

However, scientific progress has made it possible to improve the techniques of previous years and to overcome, at least in part, the limits that characterized them. In this way, contemporary microbiology can make use of molecular analysis methods, such as DNA-DNA hybridization or immunoblot techniques, genetic like metagenomics, and culture methods, such as culturomics [20]. Culturomics is a new technique that deals with the use of different culture conditions and matrix-assisted laser desorption ionization-time of flight mass spectrometry, which provides a deep increase in the number of detectable bacteria [21].

Furthermore, the treatment of oral pathologies connected to the dysbiosis of its microbiota has taken on a particular interest in the scientific community as numerous recent publications have found associations between this dysbiosis and various systemic diseases. In fact, some authors have related an increased concentration of bacteria of the *Bacillus* genus or the species *Lactobacillus reuteri* to the Autistic Spectrum Disorders and Parkinson’s disease, respectively [22,23,24]. Similar evidence was found between *Actinomyces* and *Atopobium* genera and oesophageal squamous cell carcinoma, and between *Prevotella spp.* and rheumatoid arthritis [25,26,27].

This growing interest in the oral microbiota has made it possible to raise the oral cavity to the role of a “sentinel” organ for various diseases, and to define the strictly personal concept of an “oral signature”.

Thus, some pathogenesis mechanisms that govern some systemic diseases can be triggered by bacterial populations that stay in the oral cavity. In this way, accurate analysis of a patient’s oral microbiota can immediately define their risk condition relative to a specific systemic pathology. To achieve this goal, personalized medicine can be guided by dentistry in order to create so-called “personalized dentistry”. The most frequently used therapeutic modality to eradicate a microbial component considered harmful is the prescription of an appropriate antibiotic therapy. The appropriateness of the prescription and of the posology has been the subject of recent publications that have revealed how often dental staff, unfortunately, rely on broad-spectrum antibiotics instead of prescribing antibiograms and tend to use pharmacologically inappropriate dosages [28].

Moreover, many recent publications have also reported the possibility of using biological agents to control some bacteria responsible for major oral diseases [29].

For the abovementioned reasons, a very strong effort is asked of the dental research community for adapting diagnostic and therapeutic tools to single-patient needs; research dealing with new associations between oral microflora and systemic diseases, using any type of innovative microbiological diagnostic techniques, are strongly encouraged.

The final aim of this editorial and of the whole *Special Issue* is to stimulate any type of research aimed at discovering more associations between oral bacteria and systemic diseases, at evaluating the oral bacterial response to specifically addressed therapeutic approaches, and at prospectively investigating short-term and long-term behavior of systemic diseases.

The accepted topics include all branches of internal medicine and microbiology, along with new methods for the analysis and collection of OM’s samples. Also, studies or literature reviews wishing to expand the existing sample for any previously demonstrated association will also be welcomed. Priority will be given to research concerning new therapeutic approaches or hypotheses for hardly treatable diseases. Multidisciplinary research is strongly encouraged, especially in the field of microbiology and internal medicine.
